# Behavioral Cost & Overdominance in *Anopheles gambiae*

**DOI:** 10.1371/journal.pone.0121755

**Published:** 2015-04-01

**Authors:** Malal M. Diop, Nicolas Moiroux, Fabrice Chandre, Hadrien Martin-Herrou, Pascal Milesi, Olayidé Boussari, Angélique Porciani, Stéphane Duchon, Pierrick Labbé, Cédric Pennetier

**Affiliations:** 1 MiVEGEC (UM1-UM2-CNRS 5290-IRD 224), Institut de Recherche pour le Développement (IRD), Cotonou, Bénin; 2 Centre de Recherche Entomologique de Cotonou, Cotonou, Bénin; 3 MiVEGEC (UM1-UM2-CNRS 5290-IRD 224), Institut de Recherche pour le Développement (IRD), Montpellier, France; 4 ISEM (CNRS 5554-UM2), Montpellier, France; University of Tours, FRANCE

## Abstract

In response to the widespread use of control strategies such as Insecticide Treated Nets (ITN), *Anopheles* mosquitoes have evolved various resistance mechanisms. *Kdr* is a mutation that provides physiological resistance to the pyrethroid insecticides family (PYR). In the present study, we investigated the effect of the *Kdr* mutation on the ability of female *An*. *gambiae* to locate and penetrate a 1cm-diameter hole in a piece of netting, either treated with insecticide or untreated, to reach a bait in a wind tunnel. *Kdr* homozygous, PYR-resistant mosquitoes were the least efficient at penetrating an untreated damaged net, with about 51% [39-63] success rate compared to 80% [70-90] and 78% [65-91] for homozygous susceptible and heterozygous respectively. This reduced efficiency, likely due to reduced host-seeking activity, as revealed by mosquito video-tracking, is evidence of a recessive behavioral cost of the mutation. *Kdr* heterozygous mosquitoes were the most efficient at penetrating nets treated with PYR insecticide, thus providing evidence for overdominance, the rarely-described case of heterozygote advantage conveyed by a single locus. The study also highlights the remarkable capacity of female mosquitoes, whether PYR-resistant or not, to locate holes in bed-nets.

## Introduction

In an attempt to separate the hungry malaria mosquito female from its human host, a physical and chemical barrier was introduced: the PYR ITN [1,2]. The on-going extensive distribution of ITNs aims to reach universal coverage in endemic countries [2]. Because ITNs are so effective at killing mosquitoes, and because ITNs can only be treated with PYRs, specific responses have evolved in mosquito populations to confer either behavioral or physiological insecticide resistance to these chemicals [3–7]. The most widespread physiological PYR-resistance mechanism among mosquito vectors is the target-site L1014F mutation of the voltage-gated sodium channel gene, named *Kdr* mutation. The mutated form decreases the affinity between the PYR molecule and the voltage-gated sodium channel, leading to a resistance phenotype that allows mosquito to survive contact with ITN [6,8]. The impact of this mutation on the host-seeking behavior of mosquito vectors has been largely overlooked. One especially important component of host-seeking behavior, particularly in the context of widespread ITN use, is the mosquitoes’ ability to locate and penetrate weaknesses- i.e., holes- in damaged bed nets in order to reach the human host and be able to reproduce.

We thus investigated how genotype at the *Kdr* L1014F locus (hereafter indicated as SS = susceptible homozygotes; RR = resistant homozygotes; RS = heterozygotes) affected the ability of *An*. *gambiae s*.*s*. females to find a hole in a piece of net (either untreated or treated). Females sharing the same genetic background with only the *Kdr* locus altered [9] were individually video-tracked in a wind tunnel containing an attractive odor plume orientating the mosquitoes toward a guinea pig bait. The wind tunnel consisted of two chambers separated by a holed net (S1 Fig.). Trials were recorded as successful if the mosquito passed through the hole from the first chamber (C1) to the second chamber (C2) within a 60 min assay.

## Results

A first, surprising result was that almost two-thirds of mosquitoes found the 1cm diameter hole that would have allowed them to reach their blood meal (overall success rate = 62.6%, N = 376/601, binomial 95% confidence interval CI [58.6–66.3]) in a mean time of 666.0s [588.8–742.5] (S2 Fig. panel F), regardless of the net treatment or the genotype. However, the *Kdr* genotype had a major effect on this success.

### Cost of the homozygous resistant genotype for the *Kdr* locus

**Fig 1:**
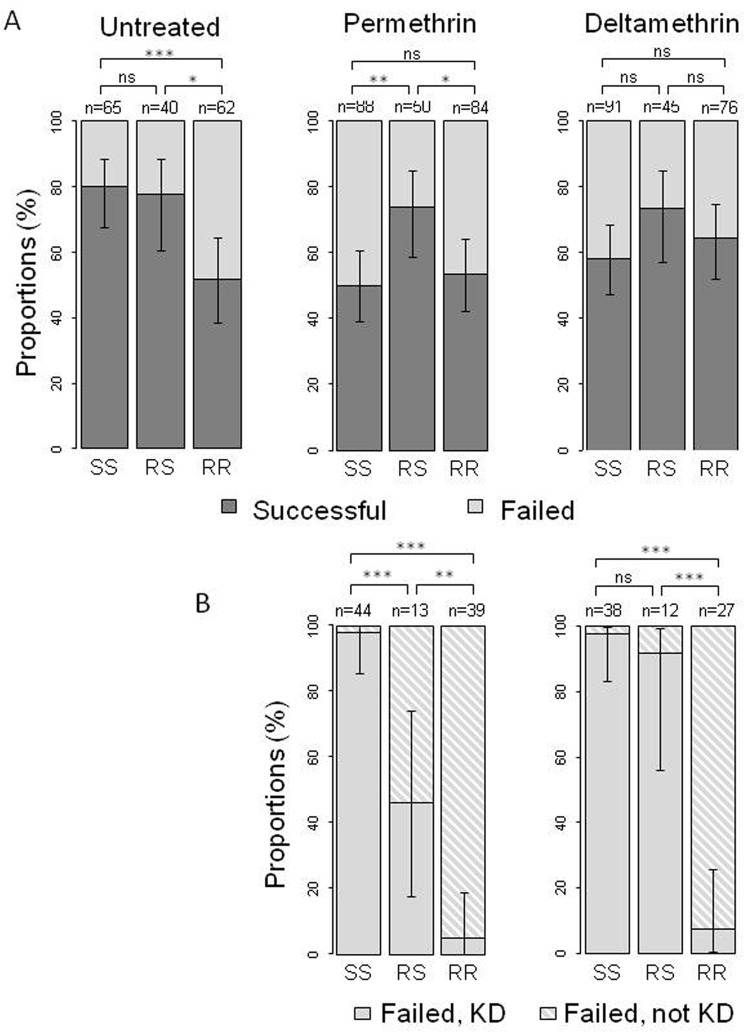
A- Proportions of Anopheles females of each *Kdr* genotype successfully penetrating a 1cm hole in (i) an untreated net, (ii) a permethrin-treated Olyset Net, and (iii) a deltamethrin-treated PermaNet 2.0. B- Proportions of knocked-down (KD) females among the failed when faced with ITN (permethrin-treated Olyset Net on the left, deltamethrin-treated PermaNet 2.0 on the right), for each *Kdr* genotype (untreated net is not presented since no mosquito from any strain presented the KD phenotype during those exposures). The number of mosquitoes tested for each genotype (SS, RS and RR: homozygous susceptible, heterozygous, and homozygous resistant for the *Kdr* mutation, respectively) is indicated. Error bars represent the 95% binomial confidence intervals for the different proportions. Significance of the different tests is indicated (^NS^ p>0.05, * p<0.05, ** p<0.01, *** p<0.001).

With an untreated holed net (UTN), the proportion of successful mosquitoes was significantly higher for both SS and RS genotypes compared to RR (binomial model odds ratios: OR_SS-RR_ = 3.75 [1.74–8.44], p = 0.0009; OR_RS-RR_ = 3.23 [1.36–8.23], p = 0.0102), while not differing significantly from each other (OR_RS-SS_ = 1.16 [0.43–3.01], p = 0.75) (Fig. 1A, left panel). The lower performance of the mutant homozygotes in the untreated net environment thus revealed a recessive behavioral cost of the *Kdr* mutation.

Analysis of behavioral traits from video tracks with untreated nets indicated that *An*. *gambiae* mosquitoes with the RR genotype spent less time flying than those with SS (Kruskal-Wallis rank sum test, p = 0.0016; Dunn’s post tests, p<0.01), and had fewer rates of contact with the holed net compared to both the SS and RS genotypes (Poisson model Contact Rate Ratio: CRR_RR-SS_ = 0.261 [0.245–0.278], p<0.0001 and CRR_RR-RS_ = 0.187 [0.176–0.2], p<0.0001) (S2 Fig.). This suggests less efficient host-seeking behavior of RR mosquitoes relative to the SS and RS types. RR mosquitoes mean flight speed was higher than that of either SS or RS mosquitoes (Kruskal-Wallis rank sum test, p<0.0001; Dunn’s post tests, p<0.01) (S2 Fig.). Mosquito flight speed has been shown to be negatively correlated with attractive odor concentration [10–12], so that higher flight speed might be an indication of less efficient odor detection in RR compared with RS and SS mosquitoes. SS and RS mosquitoes showed similar rates of success in penetrating the net, despite significant differences in the various behavioral traits (S2 Fig. panels A and C).

### Overdominance of *Kdr* mutation under PYR pressure

The behavior of the three *Kdr* different genotypes was then analyzed in presence of the two long lasting ITN recommended by the World Health Organization. One type (Olyset Net) has 1000mg/m² permethrin incorporated into it, whilst the other (PermaNet 2.0) is coated with 55mg/m² deltamethrin.

Faced with the PermaNet 2.0 net, heterozygote mosquitoes tended to be more successful in finding the hole than either of the homozygotes (Fig. 1A, right panel), although these differences were not statistically significant (OR_RS-SS_ = 1.97 [0.92–4.43], p = 0.088; OR_RS-RR_ = 1.52 [0.68–3.49], p = 0.315). This trend was reinforced (Fig. 1A, centre panel) and differences were significant when mosquitoes were faced with the Olyset Net (binomial regression, OR_RS-SS_ = 2.85 [1.36, 6.24], p = 0.007; OR_RS-RR_ = 2.47 [1.17–5.43], p = 0.02). Heterozygote mosquitoes were better able to penetrate the net than homozygous susceptible or resistant mosquitoes, regardless the net brand (when pooling data gathered with Olyset and PermaNet 2.0; OR_RS-SS_ = 2.13 [1.30, 3.49], p = 0.0027; OR_RS-RR_ = 2.42 [1.47, 4], p = 0.0005), supporting the heterozygote advantage hypothesis. There was no significant difference between homozygous susceptible or resistant mosquitoes regardless the ITN brand (Olyset: OR_RR-SS_ = 1.15 [0.63 2.11], p = 0.64; PermaNet 2.0: OR_RR-SS_ = 1.30 [0.70 2.45], p = 0.41) (Fig. 1A, center and right panels).

To further quantify this heterozygote advantage, we computed the proportion of success *p*_*i*_ as a proxy of the relative fitness (*w*_*i*_) of each genotype *i* for each treatment, as *w*_*i*_ = *p*_*i*_ */p*_*SS*_ (thus *w*_*SS*_ = 1, as reference). Moreover, by decomposing the relative fitness as *w*_*SS*_ = 1, *w*_*RS*_ = 1*+hs* and *w*_*RR*_ = 1*+s*, we were able to estimate selection (*s*) and dominance (*h*) coefficients in the different treatments. We confirmed that in the absence of PYR the R allele is deleterious (*s* = -0.35, 95% confidence interval [-0.18, -0.49]), and that this cost is recessive (*h* = 0.09 [0.01, 0.68]). However, R is advantageous in an environment with ITNs (*s* = 0.07 [0.001, 0.43] and 0.11 [0.001, 0.40] for Olyset and PermaNet 2.0, respectively). Furthermore, in ITN trials, we always found a dominance coefficient *h* > 1, confirming the observed heterozygote advantage, although quantifying this parameter precisely is more difficult: *h* = 6.72 [1.33, >100] and 2.42 [0.001, >100], for Olyset and PermaNet 2.0, respectively.

The better performances of the heterozygotes during the experiments are explained by two antagonistic forces of selection:

- Benefit of R allele in presence of insecticide
  SS females failed to find the hole because of the fast-acting knock-down (KD) effect of the PYR insecticides: 97.7% [85.3–99.9] (43/44) and 97.4% [83.2–99.9] (37/38) of the failed SS mosquitoes were KD with Olyset Net and PermaNet 2.0 respectively (Fig. 1B, multinomial model OR_PermaNet-Olyset_ = 0.861 [0.052–14.269], p = 0.917). By contrast, almost all of the unsuccessful RR mosquitoes had resisted the KD effect (KD 5.1% [0–18.6] (2/39) and 7.4% [0.04–25.7] (2/27) with Olyset Net and PermaNet 2.0, respectively) (Fig. 1B, OR_PermaNet-Olyset_ = 1.483 [0.196–11.242], p = 0.703). For heterozygotes, the result depended on the net: less than half of the failed RS were KD with permethrin-treated Olyset Net (46.2% [17.7–73.9], 6/13), while they were more affected by the deltamethrin-treated PermaNet 2.0 (91.7% [56.1–99.6], 11/12) (Fig. 1B, OR_PermaNet-Olyset_ = 12.911 [1.264–131.878], p = 0.031). This difference may be due to the difference in insecticide molecule, concentration and/or availability on the net fiber.
- Cost of RR in absence of insecticide.
  Because of the cost carried by RR mosquitoes, SS and RS mosquitoes showed higher success rates in penetrating a 1cm hole in an untreated net (Fig. 1A). Moreover, the performance of RS and RR mosquitoes in finding the hole were not altered by the insecticides (binomial model, p>0.05), while SS mosquitoes' success rate was indeed reduced by 30% [14.5–45.5] and 21.8% [6.8–36.7] with Olyset Net and PermaNet, respectively (binomial model, OR_UTN-Olyset_ = 4 [1.96–8.61], p<0.001; OR_UTN-PermaNet_ = 2.87 [1.4–6.16], p = 0.005) (Fig. 1A).

Overall, the balance between the two antagonistic selection pressures, a negative influence of *Kdr* mutation on individuals’ ability to find the hole on one hand, and the benefit for resistance to KD on the other, was most favorable to heterozygotes, providing evidence for an overdominant effect at the *Kdr* locus on this behavioral trait.

## Discussion

Insecticide resistance mechanisms are adaptations selected by challenging environmental conditions. The *Kdr* mutation is an example of a specific amino acid change at a unique position of the voltage-gated sodium channel that confers resistance to organochlorine and PYR insecticide classes in a major malaria vector in Africa, *Anopheles gambiae s*.*l*. [13]. PYR pressure can affect the mosquitoes at different stages of their life cycle: larva (contaminated breeding sites) and adult, both during resting (insecticide residual spraying) and host-seeking (ITN) periods. At these different stages, the *Kdr* mutation can allow survival, and thus reproduction, in presence of insecticides (selective advantages). However, it also imposes deleterious side-effects (selective costs), revealed in absence of insecticides. Conventional tests used to evaluate the insecticide effects on susceptible and resistant mosquitoes rely on forced and prolonged contact of the mosquitoes with the insecticide [14]. The results of these tests summarize the selection processes occurring at both the larval and adult stages of the mosquito's life and are meant to reflect the levels of resistance in the local mosquito population. Because of the higher resistance of the RR genotype, and if the mutation induces no fitness cost, prolonged insecticide selection in a population should lead to fixation of the *Kdr* mutation beyond the treated population [15]. However, as pointed out by Lynd et al. [16], there is a serious lack of evidence of *Kdr* mutation fixation in wild *Anopheles* populations, even in areas with high insecticide pressure (either from agriculture or from public health programs). Thus, they hypothesized that a fitness cost associated with the *Kdr* mutation explained the absence of fixation [16]. Such costs have been documented in *Culex quinquefasciatus* (through life history trait experiments) [17], however, none have been reported so far in *An*. *gambiae*. Interestingly, Lynd et al. [16] also suggested that the balance of advantages and costs could lead to overdominance, in which case the heterozygotes would be fitter than the SS and RR homozygotes [18–21].

Our study provides the first evidence of both a behavioural cost associated with the *Kdr* allele that conveys pyrethroid and DDT resistance in *An*. *gambiae*. Importantly, this evidence comes from an experimental set-up in which mosquito contacts with insecticide were unforced, and thus could be interrupted, similar to the situation in natural settings.

We first noted that the host-seeking performance was reduced in females homozygous for the resistance *Kdr* allele (RR) in the absence of insecticide. The RR females are less apt at finding the hole in the net to reach their blood meal. This is the first evidence of behavioral costs associated with this mutation. It suggests a deficiency in the nervous system of RR females. The voltage-gated sodium channel indeed plays a central role in message propagation in the nervous system. The *Kdr* mutation enhances closed-state inactivation of nerves, meaning that more stimulation is required before nerves fire and release acetylcholine into the synaptic cleft, relative to susceptible individuals [22]. Consequently, the *Kdr* mutation probably affects several behavior-related nervous pathways [23]. In *Kdr* resistant *Heliothis virescens* moths, pharmacological and biophysical properties of sodium channels were found to cause sluggish neural activity in the absence of PYR, and were characterized by decreased cellular and behavioral excitability of sodium channels [24]. Further physiological and behavioral investigations are underway to better understand the physiological processes underlying the behavioral changes we report here.

A second finding is that, while still partially resistant to the insecticide, the heterozygous females are not affected by the cost observed in RR females. This is evidence for heterosis, or hybrid vigor, in which the product of a cross is superior to either parent [25]. One of the modalities of heterosis is overdominance, the superior fitness of the heterozygous genotype over both homozygotes [26], though reports suggesting heterozygote advantage for single gene mutations are rare and controversial. Interestingly, the majority of the few examples came from the study of resistance to infectious diseases, such as the major histocompatibility complex in vertebrates; in insects, one of the best examples is the alcohol dehydrogenase (Adh) locus in *Drosophila melanogaster* [21]. Studying contemporaneous heterozygote advantage implies fulfillment of three criteria: i) identifying genes under selection, ii) establishing relative fitness and iii) understanding the selection mechanism [21]. The present study fulfills these criteria and, thus, provides an unambiguous new example of overdominance. A single substitution in the gene encoding the voltage-gated sodium channel (*Kdr*) indeed provides heterozygotes with resistance to the KD effect of PYR higher than susceptible homozygotes SS, while imposing little cost, if any, as compared to the decreased host-seeking success seen in resistant homozygotes RR. Compared to the homozygotes, the RS genotype maintains a better balance between the antagonistic selective pressures to survive insecticide exposure while performing a complex behavior.

Interestingly, overdominance is favorable for the evolution of new resistance alleles in the form of heterozygote duplications (i.e. duplications in which the duplicates are different alleles [27–29]). An advantageous heterozygous genotype bears a segregation cost, as only half of two heterozygotes' progeny will bear this fitter genotype. A duplication associating both alleles on the same chromosome would allow this advantageous genotype to fix by eliminating this segregation cost. A similar heterosis situation is probably responsible for the selection of duplications of the *ace-1* gene (encoding the target of organophosphorous insecticides) in both *Cx*. *pipiens* and *An*. *gambiae* [30–33]. With one susceptible and one resistance allele in tandem on the same chromosome, individuals with the duplication have fitness similar to that of heterozygotes (resistance and reduced cost [29]); such duplication allows the fixation of this heterozygote advantage in a population [30]. The overdominance at the *Kdr* locus thus provides ground for similar evolution. Interestingly, a study of *An*. *gambiae Kdr* resistance by Pinto et al. [34] in Gabon showed a significant excess of the heterozygote genotype, which could be a sign of the presence of gene duplication for *Kdr*, as was shown in the case of *ace-1* [30,33,35].

In a more applied perspective, our work highlights the overall high performance of all genotypes in the trials: our results confirmed the remarkable ability of both susceptible and resistant mosquitoes to find the only way through a bednet. These observations are in agreement with previous experimental hut studies on the blood feeding rates of *An*. *gambiae* (see review [36]). The *Kdr* resistance currently at high frequencies across much of Africa is only one of the mechanisms conferring resistance to insecticides. The impact of such insecticide resistance mechanisms on behavior and/or infection by *Plasmodium spp*. is of crucial interest [9,37,38]. A multi-disciplinary approach is needed to study in depth the complex interactions among mosquito behavior, parasite infection and human-made insecticidal barriers, with the objective of designing innovative tools that can more specifically target resistant and infectious mosquitoes [39,40].

Our study highlights the importance of behavioral studies for developing a full understanding of the evolution of insecticide resistance and its impacts. By modulating host-seeking behavior, insecticide resistance can affect the vectorial capacity of female mosquitoes. Given the ability of heterozygous mosquitoes in particular to readily overcome the barrier of a damaged ITN, the effects of insecticide resistance on host choice and biting behavior remain to be investigated.

## Experimental Procedures

### Mosquito strains and rearing

PYR insecticides target the voltage-gated sodium channel on the insects’ neurons. Non-synonymous mutations in this target site that cause resistance to insecticides are often referred to as knock-down resistance mutations (*Kdr*). These alleles confer the ability to survive prolonged exposure to insecticides without being ‘knocked-down' [6]. The substitution of a leucine by a phenylalanine at codon 1014 (L1014F) is the most common sodium channel mutation, associated with PYR resistance in African malaria vectors [41].

Two strains of *An*. *gambiae s*.*s*. were used. One is the insecticide susceptible strain Kisumu (VectorBase, http://www.vectorbase.org, KISUMU1), isolated in Kenya in 1975. This strain is susceptible and homozygous (SS) for the L1014 codon. The second strain named Kdrkis is resistant to PYR and homozygous (RR) for the L1014F *Kdr* mutation. Kdrkis was obtained by introgression of the L1014F mutation into the Kisumu genome through repeated backcrosses [9]. Heterozygous individuals (RS) were obtained through more than 15 crosses of Kisumu SS females with Kdrkis RR males. The three genotypes thus share a common genetic background for most of their genome [9].

The genotype of both susceptible and resistant strains are confirmed every 3 months by PCR following standard operational procedures of a WHO collaborating centre. For the present study, Kisumu and KdrKis strains were checked by PCR (for the *Kdr* and *ace-1* mutations) before the beginning of the behavioral assays (July 2012) and after the end of the study (May 2013) confirming that both strains were respectively homozygous susceptible and resistant for *Kdr*.

The mosquitoes were reared at 27 ± 1°C, 60–70% R.H. under 16:8h L:D photoperiod at the insectaries of the Institut de Recherche pour le Développement (IRD) in Montpellier, France. Adults were fed with a 10% glucose solution and received a blood meal twice a week. Gravid females laid eggs on cups placed inside mesh-covered cages. Eggs were dispensed into plastic trays containing de-ionized water. Larvae were kept in these trays and fed with TetraMin fish food. Pupae were removed daily and allowed to emerge inside 50x50x50cm cages. Adult females used to generate these lines were fed with rabbit blood.

Mosquitoes used in the experiments were 7–8 days old females that had never received a blood meal and were deprived of sugar the night before testing. The temperature of the experimental room was maintained at 27 ± 1°C and 60–70% R.H.

### Experimental setup

Experiments were conducted in a wind tunnel (40x13x13cm), divided into two chambers of equal dimensions separated by a piece of netting (treated with insecticide or untreated) with a 1cm diameter hole in its center (HN) (S1 Fig.). Three types of holed nets were tested in this study: untreated polyester net, Olyset Net (incorporated with 1000mg/m² of permethrin), and PermaNet 2.0 (coated with 55mg/m² of deltamethrin). The chambers (C) were numbered 1 and 2, respectively. The tunnel was made of foam board with a white opaque Plexiglas floor and a removable transparent Plexiglas roof. The ends of the chambers were screened with untreated net (NS) prevented the mosquitoes from escaping. The airflow entered the tunnel *via* a 10 cm diameter circular opening covered with an untreated net screen that acted as a diaphragm to regulate airflow in the tunnel at 16±3 cm.s^-1^.

The tunnel was softly illuminated by 12 blue LEDs (450nm) from 83cm underneath. Illumination inside the tunnel was 186.66 10^-4^ mW/cm^-2^.

The tunnel was completed by a glass cage (GC; 60x26x26cm), which held the attractive guinea pig bait (able to move in a limited area in the upper part of the cage) and a fan aimed directly down the tunnel.

Mosquitoes were released individually for each trial. The trial was replicated for each genotype and treatment. In order to get enough replicates for the analysis of the performances, a minimum of 40 mosquitoes successfully passing through the piece of net was required. The number of replicates range from 40 to 91 depending of the treatment and genotype. Each mosquito was filmed during 60 min maximum using a Sony Digital HD Video Camera (HDR-XR550), placed 50cm above the tunnel. The camera was connected to a computer in an external room from where the assay was controlled in real-time. Recording was stopped when the mosquito passed through the hole to chamber 2. MPEG-2 videos (PAL video: 720x576 pixels at 25 frames/s) were analyzed using Ethovision XT software (v.7, Noldus Information Technology, Wageningen, The Netherlands). During the trials, the mosquito was recorded as successful if it passed through the holed net to reach the upwind chamber and unsuccessful otherwise (i.e. it was still in the downwind chamber after the 60 min). Because ITN can induce a fast-acting effect known as Knock-down (KD), unsuccessful mosquitoes were recorded as KD if they were lying on their side or back with none of their tarsi in contact with the floor, or otherwise alive. Moreover, the following behavioral variables were measured in chamber 1 for the assay duration (60min or until the mosquito passed through the hole): (1) time spent on the walls (except the holed net) of chamber 1, (2) time spent on the holed net, (3) number of contacts with the holed net, (4) flight time, (5) mean flight speed, and (6) elapsed time before passing through the hole (if successful).

During the setting-up phase of each experiment, latex gloves were used to avoid any contamination with human skin odors. Mosquitoes were released individually from an opening (1cm diameter) at the downwind extremity of one of the tunnel walls. Cotton was used to plug the hole after releasing.

### Statistical analysis

All statistical analyses were conducted using the software R version 3.0.2 [42] with the additional nnet, pgrmess and spaMM packages [43–45].

### Performances

We analyzed the performance (i.e. probability of passing through the holed net) using a binomial logistic model with *Kdr* genotypes (SS, RS or RR), treatments of the holed net (untreated, Olyset Net or PermaNet) and interactions as explanatory variables. The model was written as follow:

$$\text{logit}\left(P\left(y=1\right)\right)=\beta_{0}+\beta_{i}^{Genotype}+\beta_{k}^{Treatment}+\beta_{i}^{Genotype}\times \beta_{k}^{Treatment}$$,

where$\;\beta_{i}^{Genotype}$denotes the effect on the logit of classification in category *i* (SS, RS or RR) of Genotype and $\beta_{k}^{Treatment}$denotes the effect of classification in category *k* (untreated, Olyset Net or PermaNet) of Treatment. Each combination of categories *i* and *k* of the explanatory variables was successively used as reference class to allow multiple comparisons among genotypes and treatments. Odds ratios and their 95% confidence intervals were computed. We calculated binomial confidence interval of the proportions of successful mosquitoes using Wilson's score method [46] with a continuity correction [47].

The selection parameters *h* (for dominance) and *s* (for selection) determine the proportion *p* of successful mosquitoes for the different genotypes in each trial, which are estimated by a binomial generalized linear model with predictor logit (*p*) = *a*_*g*_ for the three genotypes *g* = SS, RS, RR. *h* and *s* are complex functions of the three *a*_*g*_ coefficients. For simplicity, we therefore randomly generated *a*_*RS*_ and *a*_*RR*_ values (100,000 such pairs in a uniform distribution), and for all such pairs we fitted *a*_*SS*_ and plotted the attained likelihood against the corresponding *h* or *s* values. The upper boundary of either cloud of points is the profile likelihood for either parameter, from which maximum likelihood estimates and likelihood ratio confidence intervals were computed.

A multinomial logistic model with 3 possible outcomes (successful, unsuccessful alive or unsuccessful KD) was used to compare the proportions of KD relative to the unsuccessful mosquitoes among genotypes and between insecticidal treatments (Olyset Net or PermaNet). The multinomial model allowed us to take into account the proportion of successful mosquitoes in the analysis. Odds ratios and their 95% confidence interval were computed. We calculated multinomial confidence intervals for the proportions of KD using the method by Sison and Glaz [48] (R package "MultinomialCI").

### Behavioral variables recorded using video analyses

The number of contacts with the holed untreated net per time unit was compared among genotypes using a Poisson model with the log of the video duration (i.e. the elapsed time before the mosquito passed through the hole and 60min for successful and unsuccessful mosquitoes, respectively) as an offset.

Proportion of flight time, mean flight speed, proportions of time spent on the holed net and on the walls of chamber 1 were not normally distributed and were therefore compared among genotypes using Kruskal-Wallis tests followed by Dunn’s post-hoc tests [45,49].

For successful mosquitoes, the time needed to pass through the hole in the untreated net was also compared among genotypes using a Kruskal-Wallis tests followed by Dunn’s post-hoc tests.

## Ethical Considerations

The IRD lab where the experiments were run received the approval from the animal care and use committee named “Comité d’éthique pour l’expérimentation animale; Languedoc Roussillon” (CEEA-LR-1064 for guinea pigs and CEEA-LR-13002 for the rabbits).

## Supporting Information

S1 Fig Panel A. A wind tunnel to study the ability of malaria vector mosquitoes to pass through a holed net. C1: Chamber one (release chamber); C2: Chamber 2; GC: Glass cage receiving the guinea pig bait; RO: Release opening; NS: Net screens; HN: Holed net. Panel B. Photo of the experimental setup.
(TIF)

S2 Fig Tukey’s boxplots of (A) contact rates, (B) proportions of flight time, (C) flight speed, (D) proportion of time spent on the holed net, (E) proportions time spent on the tunnel walls, and (F) elapsed time before passing through the hole in An. gambiae of the three kdr genotypes faced with an untreated holed net. Whiskers indicate the most extreme data that is no more than 1.5 times the interquartile range. Outliers are not shown. ns: non significant, **: p<0.01, ***:p<0.001 according to (A) a Poisson model and (B, C, D, E, F) Dunn’s post tests after a Kruskal-Wallis test.
(TIF)
