# Supplementary figures and images for: Behavioral Cost & Overdominance in Anopheles gambiae
Source: PLoS One. 2015 Apr 1;10(4):e0121755. doi: 10.1371/journal.pone.0121755 (PMC4382092; doi:10.1371/journal.pone.0121755)

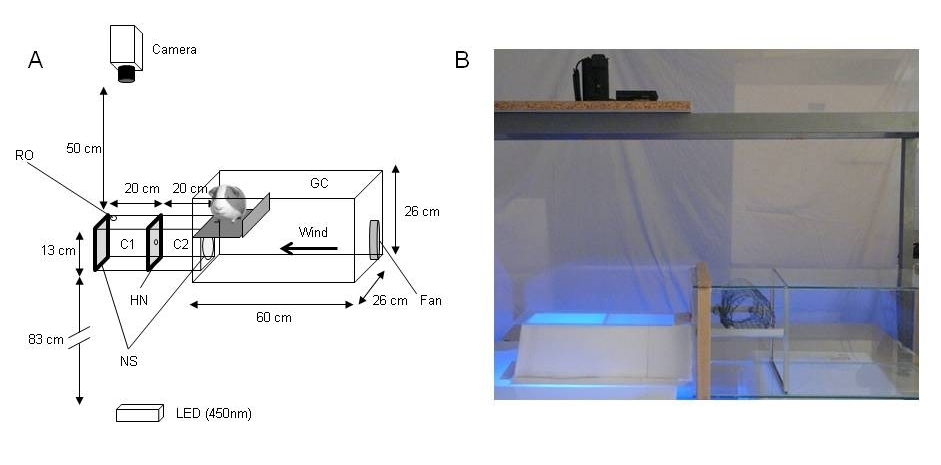

Supplement: S1 Fig — A wind tunnel to study the ability of malaria vector mosquitoes to pass through a holed net. C1: Chamber one (release chamber); C2: Chamber 2; GC: Glass cage receiving the guinea pig bait; RO: Release opening; NS: Net screens; HN: Holed net. Panel B. Photo of the experimental setup. (TIF) [file pone.0121755.s001.tif]

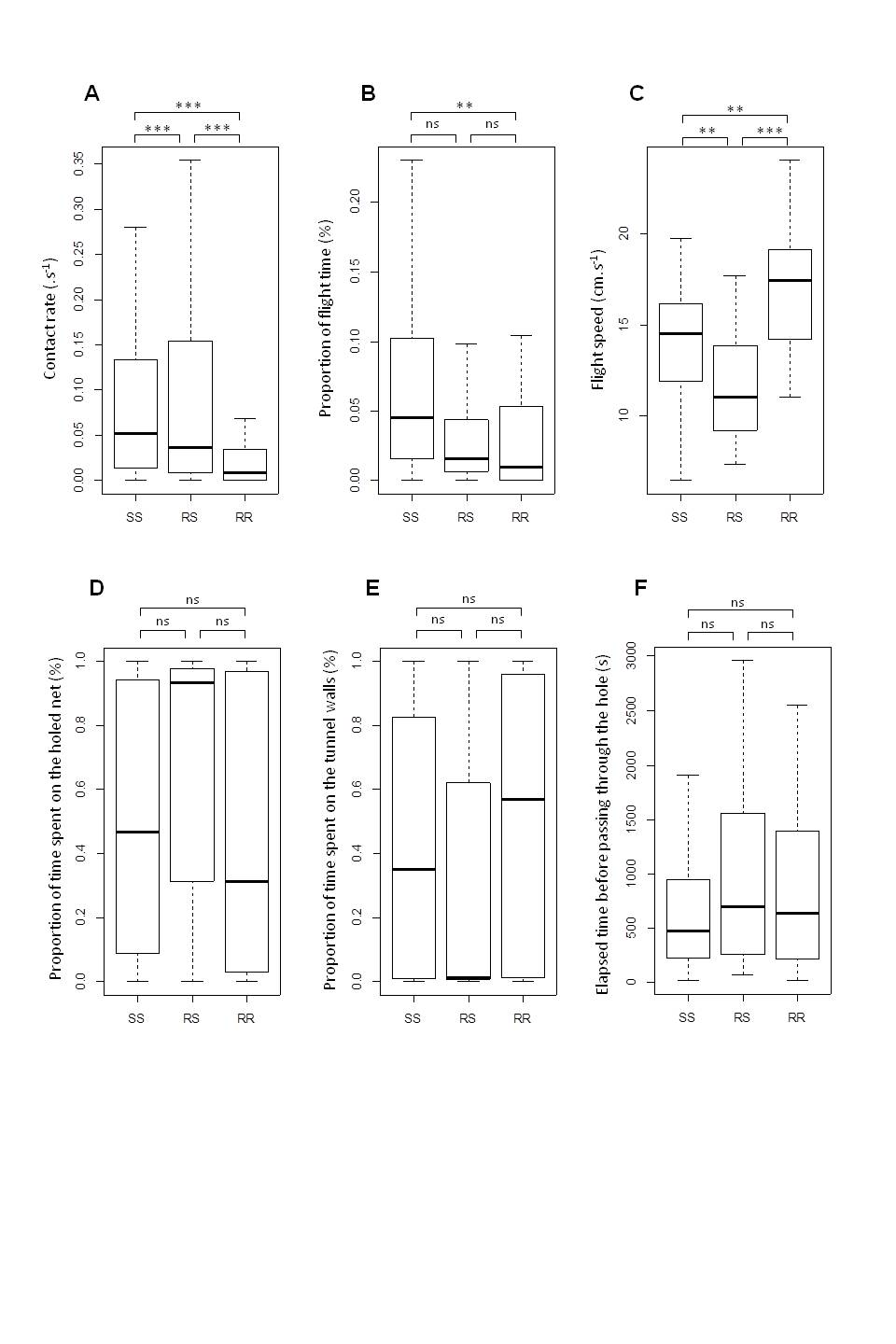

Supplement: S2 Fig — gambiae of the three kdr genotypes faced with an untreated holed net. Whiskers indicate the most extreme data that is no more than 1.5 times the interquartile range. Outliers are not shown. ns: non significant, **: p<0.01, ***:p<0.001 according to (A) a Poisson model and (B, C, D, E, F) Dunn’s post tests after a Kruskal-Wallis test. (TIF) [file pone.0121755.s002.tif]
